# Oblique Bikini Incision Versus Longitudinal Incision for Direct Anterior Approach Total Hip Arthroplasty: A Systematic Review and Meta‐Analysis

**DOI:** 10.1002/hsr2.72509

**Published:** 2026-05-10

**Authors:** Yi Leng, Jianzeng Zhang, Xin Qi

**Affiliations:** ^1^ Department of Orthopaedic Surgery The First Hospital of Jilin University Jilin China

**Keywords:** bikini incision, direct anterior approach, lateral femoral cutaneous nerve, meta‐analysis, scar cosmesis, total hip arthroplasty

## Abstract

**Background and Aims:**

Standard protocols for the direct anterior approach (DAA) typically employ a longitudinal incision, which violates Langer's lines and may compromise scar esthetics. The oblique “bikini” incision aims to improve cosmesis by following natural skin tension lines, but it raises anatomical concerns regarding iatrogenic lateral femoral cutaneous nerve (LFCN) injury. This study aimed to systematically evaluate the safety, functional, and esthetic outcomes of the bikini incision versus the traditional longitudinal incision in DAA total hip arthroplasty (THA).

**Methods:**

Adhering to the PRISMA 2020 guidelines, we searched major databases for comparative studies (both randomized controlled trials (RCTs) and observational cohorts) of bikini versus longitudinal incisions in DAA‐THA. Primary outcomes were scar cosmesis and LFCN injury incidence. Secondary outcomes included operative time and wound complications. Data were pooled using a random‐effects model, and subgroup analyses were performed to explore heterogeneity based on study design.

**Results:**

Eight studies comprising 2017 hips were included. The bikini incision demonstrated significantly superior scar cosmesis (SMD = −0.62; 95% CI [−1.00, −0.24]; *p* = 0.001) without increasing operative time (MD = −0.52 min; 95% CI [−2.62, 1.58]; *p* = 0.63). The pooled analysis showed no significant difference in overall LFCN injury risk (OR = 1.19; 95% CI [0.60, 2.36]; *p* = 0.61); however, subgroup analysis of high‐quality RCTs indicated a higher risk in the bikini group (OR = 2.15; 95% CI [1.26, 3.68]; *p* = 0.005). Additionally, the bikini group exhibited a potential trend toward reduced wound complications, though this did not reach statistical significance (OR = 0.42; 95% CI [0.15, 1.15]; *p* = 0.09).

**Conclusion:**

The bikini incision offers superior scar esthetics without prolonging operative time and demonstrates a potential trend toward minimizing wound complications. However, surgeons must remain vigilant regarding LFCN anatomy, as rigorous RCTs suggest a higher anatomical risk of nerve injury. This approach is recommended primarily for experienced surgeons who have surpassed the standard DAA learning curve.

## 1 Introduction

Total hip arthroplasty (THA) is widely recognized as a procedure of choice for end‐stage hip osteoarthritis and osteonecrosis of the femoral head [1, 2, 3]. In recent decades, the direct anterior approach (DAA) has gained substantial popularity among orthopaedic surgeons. By utilizing an intermuscular and internervous plane, the DAA theoretically minimizes soft tissue trauma and accelerates postoperative recovery compared to posterior or lateral approaches [4, 5]. Standard approaches typically employ a vertical incision vector [6]. While this vertical orientation provides effective surgical exposure, it crosses Langer's lines—the natural tension lines of the skin—perpendicularly. This misalignment creates constant lateral strain on the healing wound, which has been associated with scar widening, hypertrophy, and potential wound healing complications, particularly in patients with a higher body mass index (BMI) [7]. To address these dermatological and healing concerns, the bikini incision was proposed [8]. This technique involves an oblique incision placed within the groin crease, running parallel to Langer's lines. The modification aims to reduce skin tension, thereby improving scar cosmesis and reducing wound complications without compromising surgical exposure [9, 10].

Despite these theoretical advantages, the clinical superiority of the bikini incision remains a subject of debate. While some studies report higher patient satisfaction, others have raised safety concerns [11]. Specifically, the oblique orientation traverses the anterior thigh horizontally, potentially increasing the risk of lateral femoral cutaneous nerve (LFCN) injury due to its proximity to the nerve's branching patterns [12]. Furthermore, concerns exist that the technical demands of the bikini incision could prolong operative time [13].

Current evidence summaries have been limited by an inability to perform quantitative meta‐analyses due to heterogeneity [14]. However, the recent publication of several high‐quality randomized controlled trials (RCTs) and comparative studies necessitates an updated evaluation. Therefore, the aim of this study was to perform a systematic review and quantitative meta‐analysis to provide robust statistical evidence comparing the bikini incision versus the longitudinal incision in DAA‐THA, specifically focusing on operative time, wound complications, and LFCN injury rates.

## 2 Methods

### 2.1 Search Strategy and Selection Criteria

The reporting and methodology of this review adhered strictly to the framework outlined in the Preferred Reporting Items for Systematic Reviews and Meta‐Analyses (PRISMA) 2020 statement (Table S1). A pre‐specified protocol was registered in PROSPERO (CRD420251248277).

The search strategy was constructed using the PICO framework (Population, Intervention, Comparison, Outcome). We searched databases including PubMed, Scopus, Web of Science, and Google Scholar using terms related to “total hip arthroplasty”, “direct anterior approach”, and incision techniques such as “bikini”, “oblique”, “transverse”, or “skin crease”.

### 2.2 Eligibility Criteria

Study eligibility hinged on specific PICO definitions. We targeted patients undergoing primary THA (regardless of etiology, such as osteoarthritis or osteonecrosis), where the oblique “bikini” incision via DAA was benchmarked against the traditional longitudinal (vertical) incision. To qualify for inclusion, studies had to quantify at least one critical endpoint: LFCN safety, scar esthetics, surgical duration, or wound‐related morbidity. Conversely, we filtered out non‐comparative or non‐clinical formats (reviews, case reports, cadaveric analyses), as well as revision surgeries and non‐DAA approaches. Our retrieval strategy, anchored in the PICO framework, swept PubMed, Scopus, Web of Science, and Google Scholar using a Boolean combination of terms related to “total hip arthroplasty,” “direct anterior approach,” and incision variants (e.g., “bikini”, “oblique”, “transverse”, or “skin crease”).

### 2.3 Assessment of Study Quality and Internal Validity

Two independent investigators scrutinized the methodological rigor of the included literature. Given the mixed nature of the evidence, we tailored our assessment strategy to the study design. For randomized controlled trials, we deployed the Cochrane RoB 2 tool [15] to audit critical domains, including the randomization logic and potential deviations from intended interventions. Conversely, observational cohorts were appraised via the Methodological Index for Non‐Randomized Studies (MINORS) [16], with a specific lens on patient selection protocols and the integrity of group comparability.

### 2.4 Data Pooling and Statistical Methodology

Data synthesis and statistical analyses were conducted using Review Manager software (RevMan, version 5.4; the Cochrane Collaboration). Our statistical methodology strictly adhered to the recommendations of the Cochrane Handbook for Systematic Reviews of Interventions [17] and the SAMPL guidelines.

We stratified our effect measures based on the nature of the outcome. Dichotomous events (e.g., LFCN injury, wound complications) were analyzed using the Mantel–Haenszel method and expressed as odds ratios (ORs) with 95% confidence intervals (CIs). For continuous data, we applied the inverse‐variance method, utilizing the mean difference (MD) for uniform metrics (e.g., operative time in minutes) and the standardized mean difference (SMD) when different measurement scales were used (e.g., scar cosmesis scores).

Anticipating inherent clinical and methodological variability across the included studies, we utilized the DerSimonian and Laird random‐effects model for all pooled analyses [18]. Statistical heterogeneity was evaluated using the Cochran's Q (*χ* ^2^) test and quantified by the *I* ^2^ statistic, with values of 25%, 50%, and 75% corresponding to low, moderate, and high heterogeneity, respectively [19]. The overall pooled effect sizes were tested for significance using the *Z*‐test.

To dissect the potential sources of high variance, we conducted a prespecified subgroup analysis to distinguish between randomized controlled trials (RCTs) and observational cohorts. All statistical tests were two‐sided, and an a priori level of significance was set at *α* = 0.05.

## 3 Results

### 3.1 Search Results and Study Selection Flow

**Figure 1:**
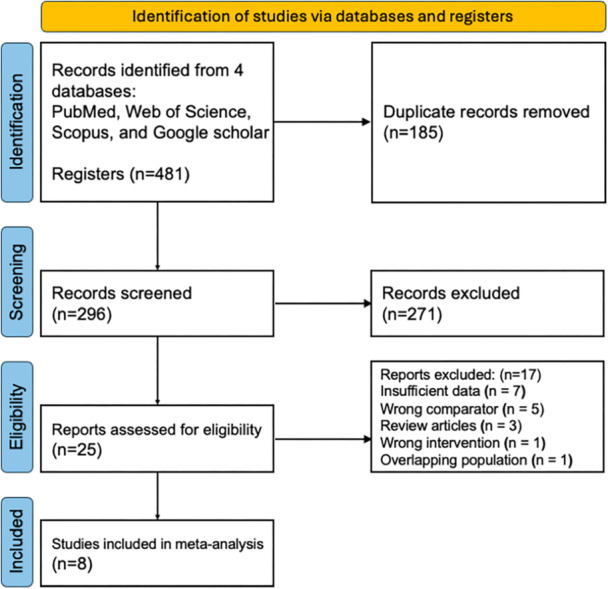
PRISMA (preferred reporting items for systematic reviews and meta‐analyses) flowchart detailing the study selection process for inclusion in the meta‐analysis.

Our initial search strategy harvested a raw pool of 481 citations across the targeted databases: PubMed (*n * = 101), Web of Science (*n *= 137), Scopus (*n *= 143), and the top 100 hits from Google Scholar. The first phase of data cleaning involved the removal of 185 redundancies, leaving a working list of 296 unique records. Subsequent title and abstract screening proved rigorous, resulting in the culling of 271 candidates that failed to align with the core inclusion criteria. This left 25 potential articles for full‐text scrutiny. Upon detailed examination, a further 17 studies were discarded, primarily due to data deficits (inconvertible outcomes for cosmesis or complications) or incompatible study architectures (such as narrative reviews or case reports). This filtration process crystallized into a final cohort of eight studies eligible for quantitative synthesis (Figure 1). Detailed baseline metrics for these included trials are cataloged in Table 1.

**Table 1:** Baseline characteristics of the included studies comparing the bikini and longitudinal incisions for direct anterior approach total hip arthroplasty (DAA‐THA).

Author, year	Country	Study design	LOE	Sample size (B/L)	BMI (B/L)	Follow‐up
Sang et al. (2021) [20]	China	RCT	I	99/96	24.5/24.8	6 months
Wang et al. (2021) [21]	China	RCT	I	49/50	25.1/24.9	12 months
Nagda et al. (2025) [22]	Canada	Prospective cohort	II	23/32	29.5/28.8	1.5–12 months
Manrique et al. (2019) [23]	USA	Retrospective case‐control	III	86/230	34.5/34.2	6 months
Leunig et al. (2018) [24]	Switzerland	Retrospective cohort	III	398/556	26/26	2–4 years
Di Martino et al. (2023) [25]	Italy	Retrospective cohort	III	52/58	26.8/27.1	25 months
Menzies‐Wilson et al. (2020) [26]	UK	Retrospective cohort	III	89/124	28/28	1 year
Wang et al. (2021) [27]	China	Retrospective cohort	III	32/43	24.3/23.8	1–3 months

Abbreviations: B/L, bikini incision/longitudinal incision; DAA‐THA, direct anterior approach total hip arthroplasty; LOE, level of evidence; RCT, randomized controlled trial.

### 3.2 Risk of Bias Assessment

**Figure 2:**
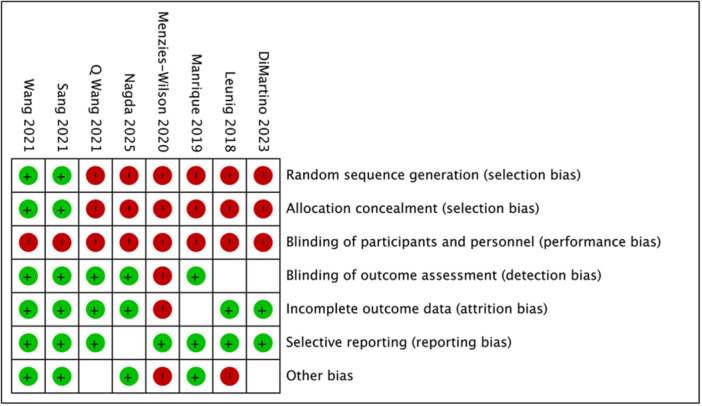
Overview of methodological quality judgments and risk of bias across the eight included studies. Green circles (+) indicate a low risk of bias, blank/yellow circles indicate some concerns or unclear risk, and red circles (−) indicate a high risk of bias for each specific domain.

The methodological quality of the seven included studies was assessed using the RoB 2 tool for RCTs and the MINORS/NOS scale for observational studies. The risk of bias summary is presented in Figure 2.

Among the two RCTs, Sang et al. was judged to be at low risk of bias, employing appropriate randomization and allocation concealment [20]. Wang et al. were rated as having some concerns regarding the measurement of the outcome, as patients were not blinded to the incision type, which could potentially influence subjective scar satisfaction scores [21].

Regarding the five observational studies, Manrique et al. demonstrated high quality due to rigorous 3:1 matching of the study cohorts [23]. In contrast, we assigned a moderate bias rating to the observational series reported by Leunig et al., Menzies‐Wilson et al., and Di Martino et al., primarily due to selection bias inherent in retrospective designs and performance bias (e.g., bikini incisions performed by more experienced surgeons) [24, 25, 26]. Notably, the pilot study by Nagda et al. was flagged for high bias potential arising from significant confounding, with a substantial gender imbalance between the groups (91% (21/23) female in the bikini group versus 50% (16/32) in the control group) [22].

### 3.3 LFCN Injury

**Figure 3:**
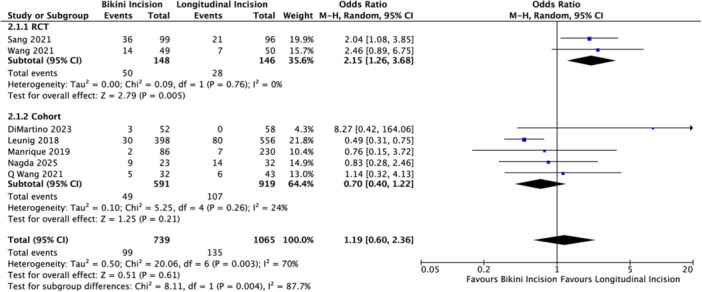
Forest plot comparing the risk of LFCN injury between the bikini incision and longitudinal incision groups. The size of the blue squares is proportional to the statistical weight of each study. Horizontal lines represent 95% confidence intervals CIs. The black diamond represents the overall pooled OR calculated using the Mantel–Haenszel (M‐H) random‐effects model.

Seven studies provided extractable data on LFCN complications [20, 21, 22, 23, 24, 25, 27]. The initial pooled analysis was equivocal, showing no clear dominance for either technique (OR = 1.19; 95% CI [0.60, 2.36]; *p*= 0.61; Figure 3). Yet, the high heterogeneity (*I* ^2^ = 70%) hinted at underlying inconsistency. Stratification by study design resolved this ambiguity, revealing a sharp discordance between methodological tiers (subgroup difference: *p *< 0.01). Specifically, high‐quality RCTs exposed a specific vulnerability: they demonstrated a more than two‐fold increase in nerve injury risk for the bikini group (OR = 2.15; 95% CI [1.26, 3.68]; *p *= 0.005). In stark contrast, observational studies failed to replicate this signal, instead showing a non‐significant trend leaning towards safety (OR = 0.70; 95% CI [0.40, 1.22]; *p* = 0.21).

### 3.4 Scar Cosmesis

Scar cosmesis and patient satisfaction quantitative data regarding scar cosmesis were available from four studies [21, 22, 23, 25], involving a total of 468 hips. Since different scoring systems were utilized (e.g., Patient and Observer Scar Assessment Scale (POSAS), SCAR score), the SMD was used for analysis.

**Figure 4:**
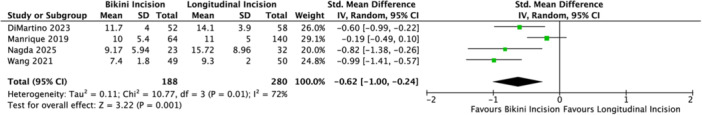
Forest plot comparing scar cosmesis scores between the bikini incision and longitudinal incision groups. The size of the green squares indicates the statistical weight of the study, and horizontal lines denote 95% CIs. The black diamond indicates the pooled SMD utilizing an inverse‐variance (IV) random‐effects model.

Quantitatively, the bikini incision yielded a robust visual dividend. Patients consistently rated their scars more favorably compared to the longitudinal group, reflected in a substantial SMD of −0.62 (95% CI [−1.00, −0.24]; *p* = 0.001). While we observed statistical noise (*I* ^2 ^= 72%), this is a predictable artifact of pooling diverse metrics (such as the POSAS vs. SCAR systems) and varying maturation times (up to 4 years). Despite this methodological friction, the signal remained uniform: every included study pointed towards superior cosmesis. This consistency across studies strengthens the statistical signal, although variability in scoring systems and follow‐up duration should be considered when interpreting clinical relevance (Figure 4).

### 3.5 Operative Time

**Figure 5:**
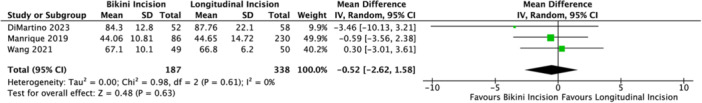
Forest plot comparing operative time (minutes) between the bikini incision and longitudinal incision groups. The size of the green squares reflects the statistical weight, with horizontal lines representing 95% CIs. The black diamond shows the pooled MD using an inverse‐variance (IV) random‐effects model.

Does the aesthetic gain of the bikini incision require a temporal sacrifice? The pooled analysis did not demonstrate a statistically significant difference in operative time between the two approaches. Synthesis of operative logs from the three reporting cohorts revealed a statistically negligible difference between techniques (MD = −0.52 min; 95% CI [−2.62, 1.58]; *p*= 0.63; Figure 5) [21, 23, 25]. Remarkably, the heterogeneity was nonexistent (*I* ^2^ = 0%), signaling perfect consistency across surgical teams. These findings suggest that, among experienced surgeons, the bikini modification does not appear to impose a measurable time burden in the included studies.

### 3.6 Wound Complications

**Figure 6:**
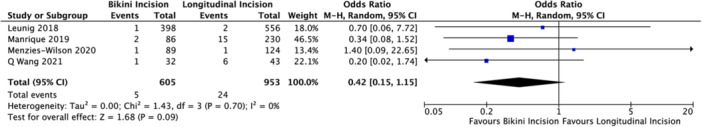
Forest plot illustrating the comparative risk of wound‐related complications between the bikini incision and longitudinal incision groups. The blue squares indicate point estimates with sizes proportional to study weights, and horizontal lines indicate 95% CIs. The black diamond depicts the pooled OR derived from the Mantel–Haenszel (M‐H) random‐effects model.

Aggregating data from four cohorts, we analyzed the incidence of wound morbidity (infection, dehiscence, and delayed healing). The pooled analysis indicated that the bikini incision demonstrated a trend toward reducing the odds of wound complications (OR = 0.42; 95% CI [0.15, 1.15]). However, because this finding did not reach statistical significance (*p*= 0.09), it must be interpreted with caution as a potential trend rather than a definitive clinical advantage. The clinical relevance of this “near‐miss” crystallizes when examining high‐risk phenotypes. As highlighted by Manrique et al., the benefit becomes statistically unambiguous in the obese population (BMI > 30 kg/m²), where the bikini technique completely eliminated delayed wound healing (0% (0/16) versus 16.6% (8/48)). This may reflect reduced skin tension and avoidance of the moist inguinal fold in patients with a larger panniculus; however, this mechanistic interpretation remains speculative (Figure 6).

### 3.7 Sensitivity Analysis

To ensure our conclusions were not fragile, we stress‐tested the stability of the pooled results via sequential “leave‐one‐out” sensitivity analyses. A specific audit was directed at the pilot study by Nagda et al., which was flagged for high risk of bias. We surgically excised this data set to see if the cosmetic advantage would collapse without it. It did not. The pooled estimate for superior scar cosmesis remained statistically immutable even after this targeted exclusion. The consistency of results across sensitivity analyses supports the robustness of the aesthetic findings and suggests that they are unlikely to be solely driven by lower‐quality studies.

## 4 Discussion

By integrating the latest wave of high‐grade evidence‐spanning both randomized trials and observational cohorts—this study supports the clinical viability of the oblique bikini modification for DAA‐THA. Our pooled analysis revealed three key findings: (1) the bikini incision offers statistically superior scar cosmesis and patient satisfaction; (2) contrary to concerns regarding technical complexity, it does not prolong operative time; and (3) it is associated with a potential trend toward a lower rate of wound complications compared to the longitudinal incision. While the risk of LFCN injury remains a subject of debate, our subgroup analysis suggests that this risk may be detection‐dependent.

The aesthetic superiority of the bikini incision is anatomically well‐founded. As described by Lemperle et al., surgical incisions placed parallel to the main folding lines (Langer's lines) result in minimal collagen disruption and reduced skin tension during healing [28]. In contrast, the traditional longitudinal incision runs perpendicular to these lines, creating lateral tension that predisposes the wound to hypertrophic scarring and widening. Our meta‐analysis confirmed this theoretical advantage with a moderate‐to‐large effect size (SMD = −0.62). Importantly, this finding proved robust in sensitivity analysis, maintaining statistical significance even after the exclusion of high‐bias studies. Furthermore, as highlighted by Nagda et al., the scar is easily concealed by undergarments, boosting patient confidence.

A clinically relevant finding of our study is the potential protective effect of the bikini incision against wound complications. Although the difference did not reach statistical significance in the overall pooled analysis (*p* = 0.09), the odds ratio (OR = 0.42) suggests a trend toward risk reduction. This challenges the traditional view that transverse incisions might compromise healing due to vascular disruption. On the contrary, the longitudinal incision often crosses the moist environment of the groin fold, which can harbor bacteria and lead to maceration. This mechanism is strongly supported by Watts et al., who identified obesity as a significant independent risk factor (hazard ratio 4.3) for wound complications in DAA THA, attributing this primarily to the proximity of the incision to the abdominal panniculus [29]. This aligns with the broader literature on soft tissue management in obese populations. Adipose tissue is known to be poorly vascularized and prone to dead space formation, making it particularly susceptible to ischemia when subjected to tension [30]. A longitudinal incision perpendicular to the skin tension lines experiences constant distraction forces, especially during hip flexion, which can compromise the delicate microcirculation of the healing wound edges [28]. By orienting the incision parallel to Langer's lines, the bikini approach minimizes these vector forces and avoids the “valley” of the inguinal crease‐a region often characterized by moisture accumulation and bacterial colonization (intertrigo)—thereby creating a more favorable, aerobic environment for healing. Consistent with this mechanism, Manrique et al. further demonstrated that in obese patients (BMI > 30 kg/m²), the bikini incision significantly reduced delayed wound healing from 0% (0/16) versus 16.6% (8/48) [23]. Furthermore, it is important to interpret these findings in the context of patient demographics. The protective effect of the bikini incision on wound healing was most evident in the study by Manrique et al., conducted in a Western population with a higher average BMI (mean > 30 kg/m² in the obesity subgroup) [23]. In contrast, the RCTs by Sang et al. and Wang et al. were conducted in Asian populations with significantly lower average BMIs (approx. 24–25 kg/m²), where wound complication rates were generally low in both groups [20, 21]. This disparity suggests that while the bikini incision is safe for all patients, its specific clinical advantage in preventing wound complications is likely most pronounced in patients with a larger panniculus, a phenotype more prevalent in Western cohorts. Available evidence indicates a phenotype‐specific advantage. Although observational data suggest a potential benefit in patients with higher BMI, this evidence is primarily derived from subgroup analyses and non‐randomized cohorts. Therefore, while the bikini incision may be particularly advantageous in obese patients, this hypothesis requires confirmation in adequately powered randomized trials before definitive practice recommendations can be made.

A common barrier to the adoption of the bikini incision is the perception that it is technically more demanding and time‐consuming than the standard approach. However, our meta‐analysis refutes this concern. We found no statistically significant difference in operative time between the two groups (MD = −0.52 min; 95% CI [−2.62, 1.58]; *p* = 0.63). Notably, the heterogeneity for this outcome was zero (*I* ^2^ = 0%), indicating highly consistent findings across different healthcare systems and surgeon experiences. This efficiency can be explained by the fact that the modification is limited strictly to the superficial skin and subcutaneous dissection; the deep surgical steps—including capsulotomy, femoral exposure, and acetabular preparation—remain identical to the standard DAA. It is important to acknowledge, however, that the surgeons in the included studies were likely experienced. A recent registry analysis of over 15,000 procedures by Peters et al. confirmed that the learning curve for the DAA stabilizes after approximately 100 cases [31]. Our results suggest that for surgeons who have already surmounted this learning threshold, transitioning to the bikini incision can likely be achieved with a minimal additional learning curve and no significant time penalty.

The risk of LFCN injury remains the primary concern. Our subgroup analysis revealed a divergence: RCTs utilizing rigorous ultrasound screening reported a higher injury rate in the bikini group (Sang et al.) [20], whereas observational studies reported no difference or even a protective trend (Leunig et al.) [24]. We hypothesize that this discrepancy might partially stem from a “detection bias”, although this remains speculative. High‐quality RCTs provide robust evidence that the transverse orientation carries an elevated anatomical and structural risk to the LFCN. However, when observing patient‐reported outcomes in real‐world cohorts, it is crucial to note the difference between subclinical structural injuries detected by instruments and clinically bothersome symptoms. Nevertheless, the findings from RCTs warrant heightened intraoperative vigilance. Observational studies, which rely on patient‐reported outcomes, found no significant difference in injury rates. This implies that while the transverse incision carries a higher anatomical risk, many of these injuries may be neurapraxias that do not significantly compromise overall patient satisfaction. Rudin et al. demonstrated that the LFCN often branches early over the sartorius muscle. Additional anatomical studies clarify this risk: Majkrzak et al. found the nerve typically exits within 1.5 cm of the anterior superior iliac spine, while Ray et al. noted that the nerve may pierce the sartorius muscle or fascia in up to 30% of cases, making it vulnerable during superficial dissection [32, 33]. Furthermore, Ropars et al. described a danger zone extending up to 9 cm distal to the anterior superior iliac spine where the nerve branches fan out, warning that transverse incisions geometrically increase the probability of intersection. Ropars et al. also emphasized that retractor placement in the intermuscular space can cause traction injury to the femoral branch even without transection [34]. Thus, while subclinical injury (detectable by instruments) may be more frequent, it does not necessarily translate into clinically bothersome symptoms for patients.

Surgeons must remain aware of potential pitfalls not captured by standard metrics. Recently, Banasiak et al. reported a high incidence of transient postoperative lymphedema with the bikini approach, likely due to the disruption of superficial lymphatic channels running parallel to the inguinal ligament [35]. Another limitation concerns the duration of follow‐up. Most included studies reported short‐term outcomes, typically ranging from 6 weeks to 1 year. While Leunig et al. provided medium‐term data (2–4 years), the long‐term evolution of the bikini scar—specifically regarding potential widening or positional changes due to skin aging and gravity over a decade—remains to be fully elucidated [24]. Third, most procedures in the included studies were performed by high‐volume, experienced DAA surgeons. Our findings regarding operative time and safety may not be generalizable to surgeons who are still in the early phase of their DAA learning curve. Fourth, we must acknowledge the inherent susceptibility of the included observational studies to selection bias. There is a potential for “confounding by indication”, where surgeons may have preferentially selected “ideal” candidates (e.g., thinner patients or those with favorable pelvic anatomy) for the bikini incision while reserving the longitudinal approach for more complex cases. This could artificially skew outcomes such as operative time and wound complications in favor of the bikini group. Finally, regarding the specific concern of LFCN injury, although we identified a divergence between anatomical risk and clinical symptoms, the long‐term natural history of these injuries remains unclear. Future studies with extended follow‐up (> 2 years) are necessary to determine whether the subclinical neural deficits detected in RCTs resolve spontaneously through re‐innervation or evolve into chronic meralgia paresthetica. Similarly, the permanence of subclinical LFCN injuries detected in RCTs requires longer observation periods to assess their true clinical impact. Furthermore, as noted by Zappley et al., the bikini incision is non‐extensile; in cases of intraoperative femoral fractures or early revisions, extending a transverse incision is technically challenging and may require plastic surgery techniques, whereas a longitudinal incision can be easily extended distally [36, 37]. Therefore, the bikini incision should be reserved for primary, uncomplicated cases performed by experienced surgeons.

Based on these findings, future research should prioritize two key areas. First, prospective longitudinal studies with extended follow‐up (minimum 2 years) are imperative to delineate the natural history of LFCN injury. Specifically, distinguishing between transient neurapraxia and chronic meralgia paresthetica is crucial for accurate patient counseling. Second, given the disparate wound complication rates observed between Asian and Western cohorts, multi‐center trials stratified by BMI classes and ethnic backgrounds are warranted. Such studies would help validate whether the protective benefit of the bikini incision is strictly phenotype‐dependent, thereby refining patient selection criteria.

## 5 Conclusion

Current evidence suggests that the oblique bikini incision is a viable alternative to the standard longitudinal DAA, demonstrating a statistically significant improvement in scar cosmesis without significantly prolonging operative time. Furthermore, it demonstrates a potential trend toward minimizing wound complications, particularly in obese patients. However, these aesthetic benefits must be carefully weighed against an elevated anatomical risk of LFCN injury, as highlighted by high‐quality randomized data. Given these neurovascular concerns and the limited extensibility of the transverse incision in complex cases, we recommend a cautious adoption strategy. The bikini modification is best reserved for primary, uncomplicated cases performed by surgeons who have successfully navigated the conventional DAA learning curve (typically > 100 cases).

## Author Contributions

Conceptualization and study design were spearheaded by Yi Leng and Xin Qi. The protocols for literature screening and data extraction were executed by Yi Leng alongside Jianzeng Zhang. Formal statistical synthesis was conducted by Xin Qi. The original draft was prepared by Yi Leng. All listed authors have reviewed the final text and consented to its publication.

## Funding

The authors have nothing to report.

## Ethics Statement

Ethical approval was waived for this investigation, as it exclusively analyzes previously published, anonymized data and does not involve direct interaction with human or animal subjects.

## Consent

Not applicable, given the systematic review nature of this study, utilizing secondary data.

## Conflicts of Interest

The authors declare no conflicts of interest.

## Transparency Statement

The corresponding author, Xin Qi, affirms that this manuscript is an honest, accurate, and transparent account of the study being reported; that no important aspects of the study have been omitted; and that any discrepancies from the study as planned (and, if relevant, registered) have been explained.

## Supporting information

Supporting File

## Data Availability

The data that support the findings of this study are available from the corresponding author upon reasonable request.
